# Association of personality traits with polypharmacy among community-dwelling older adults in Japan: a cross-sectional analysis of data from the SONIC study

**DOI:** 10.1186/s12877-022-03069-5

**Published:** 2022-04-28

**Authors:** Yuko Yoshida, Tatsuro Ishizaki, Yukie Masui, Yasumichi Arai, Hiroki Inagaki, Madoka Ogawa, Saori Yasumoto, Hajime Iwasa, Kei Kamide, Hiromi Rakugi, Kazunori Ikebe, Yasuyuki Gondo

**Affiliations:** 1 Tokyo Metropolitan Institute of Gerontology, 35-2 Sakae-cho, Itabashi-ku, Tokyo, Japan; 2 Keio University, 35 Shinanomachi, Shinjuku-ku, Tokyo, Japan; 3 Osaka University, 2-2 Yamadaoka, Suita, Osaka Japan; 4 Fukushima Medical University, 1 Hikariga-oka, Fukushima, Japan

**Keywords:** Polypharmacy, Neuroticism, Extraversion, Lifestyle-related disease, Psychosocial characteristics

## Abstract

**Background:**

Polypharmacy is a serious concern among older adults and is frequently related to adverse outcomes, including health problems, reduced quality of life, and increased medical expenses. Although personality traits are associated with health behaviors and diseases, the effect of polypharmacy on personality traits is unclear. Therefore, we examined the association of personality traits with polypharmacy among community-dwelling older adults.

**Methods:**

This cross-sectional study analysed data on 836 community-dwelling older adults aged 69–71 years who participated in the Japanese longitudinal cohort study of Septuagenarians, Octogenarians, and Nonagenarians Investigation with Centenarians. Polypharmacy was defined as the intake of ≥ 5 medications concurrently. Personality traits were assessed using the Japanese version of the NEO-Five-Factor Inventory (NEO-FFI). A five-factor model of personality traits, including “neuroticism,” “extraversion,” “openness,” “agreeableness,” and “conscientiousness,” was measured by the NEO-FFI.

**Results:**

The average number of medications was about 3 in both men and women. Among the participants, polypharmacy was observed in 23.9% of men and 28.0% of women. Multivariable logistic regression analysis showed that neuroticism (adjusted odds ratio [aOR] per 1 point increase = 1.078, 95% confidence interval [CI] = 1.015–1.144) in men and extraversion (aOR = 0.932, 95% CI = 0.884–0.983) in women were associated with polypharmacy.

**Conclusions:**

Higher neuroticism in men and lower extraversion in women were associated with polypharmacy. This study suggests that personality traits may be involved in the process leading to the development of polypharmacy. Information on individual personality traits may help medical professionals in decision-making regarding medication management for lifestyle-related diseases.

## Background

Most “baby boomers” will be aged > 75 years by 2025, and the number of older adults is expected to continually increase worldwide, including in Japan. Hence, it is necessary to create an environment where people can live safely in the community for as long as possible, even if medical and nursing care is required [1]. People aged > 75 years frequently experience increasing health problems and multimorbidity. The increase in multimorbidity with age leads to an increase in polypharmacy in older adults [2]. Polypharmacy is a common condition among older people, with prevalence ranging from 26.3% to 39.9% in European countries [3], Another study found the prevalence to range from 27 to 59%, in a review of 14 studies [4]. Therefore, it is recommended to use Beers Criteria [5], the STOPP/START criteria [6], and CRIME [7] to avoid inappropriate drug use and polypharmacy in each country. Therefore, polypharmacy has become a public health problem worldwide.

Polypharmacy is defined as the simultaneous use of several medications by a single individual. Previous studies have shown that polypharmacy among older adults is associated with adverse health outcomes such as falls [8, 9], reduced mobility [10, 11], cognitive impairment [11, 12], frailty [13, 14], and mortality [15, 16]. This increase in adverse health outcomes and the consequent increase in medical expenses impair the quality of life (QOL) of older adults [3]. Although multidrug therapy is an established and frequently effective treatment method, it should be used with caution in older adults with impaired physiology.

The most influential factors related to polypharmacy are older age [3, 17, 18] and the number of chronic diseases [3]. However, several other factors have also been shown to be associated with polypharmacy. For example, sociodemographic and lifestyle factors have been reported to be associated with polypharmacy. Additionally, several studies have found that low education levels [3, 17, 19], low economic status [3], physical inactivity [3], smoking [17], and drinking [18] are associated with polypharmacy.

Furthermore, psychosocial factors have been reported to be associated with polypharmacy. Previous studies have reported that self-related health [20], quality of life, and network satisfaction [3] are associated with polypharmacy. The relationship between personality traits and polypharmacy in older adults has also been reported. Personality traits are defined as “dimensions of individual differences in tendencies to show consistent patterns of thoughts, feelings, and actions” [21]. The five-factor model of personality traits consists of five major domains—neuroticism, extraversion, openness, agreeableness, and conscientiousness [22]. In addition, a previous study has shown that neuroticism is associated with polypharmacy [23]. Three possible pathways are underlying the association between personality traits and polypharmacy—lifestyle, illness, and thinking styles and attitudes. Personality traits affect an individual’s health behaviors such as smoking, alcohol intake, dietary pattern [24]. In addition, personality traits are associated with chronic diseases, for which many drugs are prescribed, such as diabetes mellitus [25], sleep disorder [26], cognitive impairment [27], and infection [28]. Personality traits are also predictors of all-cause mortality among community-dwelling older adults [29]. In addition, personality traits are associated with treatment perceptions and desires [30]. This suggests that personality traits are associated with health behaviors such as medication management and consultation behaviors, and thus, they may be involved in several processes leading to polypharmacy.

There are a limited number of studies that examined the relationship of personality traits with polypharmacy among older adults. Investigating this relationship may improve the understanding of the mechanisms underlying the high prevalence of polypharmacy among older adults and help develop more effective strategies for preventing improper medication use. In particular, personality traits may provide valuable information to determine which methods are appropriate for treatment and medical and behavioral interventions. Thus, this study aimed to explore the association of personality traits with polypharmacy among community-dwelling older adults.

## Methods

### Sample and participants

The data reported in this cross-sectional study were obtained from the first wave of the Japanese longitudinal cohort study of Septuagenarians, Octogenarians, and Nonagenarians Investigation with Centenarians (SONIC). The SONIC study used a narrow age-range cohort design. Data were collected for the 70-year-old cohort in 2010. Participants were randomly selected from the resident registry from the following urban and rural areas in Japan: Itami City, Hyogo (Western urban); Asago City, Hyogo (Western rural); Itabashi Ward, Tokyo (Eastern urban); and Nishitama County (Ome City, Okutama Town, Hinode Town, and Hinohara Village), Tokyo (Eastern rural). The participants were all volunteers living independently; no institutionalized individuals were included. A detailed description of the study design and protocol has been published elsewhere [31].

A total of 4307 residents in the 70-year-old cohort who were randomly selected from local residential registries were invited to participate in this study. The invitation letters sent out to invitees described the purposes of the SONIC study and its methodology. We asked them to send back an agreement letter, wherein invitees indicated the appropriate date and time of participation if they were interested. In total, 1000 community-dwelling older adults aged 69–71 years (479 men and 521 women) participated in the study. Of these 1000 participants, 164 participants were excluded from the analysis owing to missing medication status (*n *= 2), personality traits data (*n* = 132), subjective economic status (*n* = 5), smoking habits (*n* = 2), alcohol consumption (*n* = 18), and instrumental activities of daily living (IADL) (*n* = 5). Therefore, the data on 836 participants (397 men and 439 women) were used for this study.

Data for the SONIC study were primarily collected by administering questionnaires to participants. The questionnaires contained items designed to identify sociodemographic status, psychosocial characteristics, medical conditions, dental conditions, and lifestyles.

### Data collection

The participants were invited to the health examination survey. A questionnaire was mailed in advance to the participants’ homes and filled out by each participant. At the time of the health examination survey, missing or illogical answers to questions included in the questionnaire were checked in face-to-face interviews conducted by trained interviewers.

### Measurements

#### Polypharmacy

Participants reported their current medication status before their health examination using a questionnaire, and they brought a personal medication record to the health examination. A medication record or medicine information sheet is usually provided to patients when a physician prescribes medications. Based on the data from the questionnaire and medication record, a physician or a health professional interviewed the participants and reconfirmed their drugs, including prescription names, dosages, and duration of medications. In addition, over-the-counter drugs were not included. Polypharmacy was defined as the intake of ≥ 5 medications concurrently [15].

#### Personality traits

Personality traits were evaluated using the NEO-Five-Factor Inventory (NEO-FFI), a widely replicated model of personality [32]. We used the Japanese version of the NEO-FFI [33]. A five-factor model of personality traits, including “neuroticism,” “extraversion,” “openness,” “agreeableness,” and “conscientiousness,” was measured by the NEO-FFI. In the SONIC study, participants were asked to indicate their level of agreement on a 5-item Likert scale ranging from strongly disagree to strongly agree, with 60 statements regarding the five domains of personality traits. The total scores were calculated by summing the scores for the 12 items representing each domain after reversing negatively keyed items, resulting in variables with scores ranging from 0 to 48.

#### Other variables

The questionnaire contained items assessing demographic information, including sex, age, subjective economic status, and participants’ health-related behaviors, including smoking habits (currently or not) and alcohol consumption (currently or not).

Body mass index (BMI) was calculated as weight (in kilograms) divided by height (in meters squared), measured during health examinations. Participants’ chronic disease history was evaluated based on questions regarding a history of hypertension, diabetes mellitus, hyperlipidemia, cancer, coronary heart disease, cerebrovascular disease, tuberculosis, pneumonia, gastric or liver disease, renal or prostate disease, osteoporosis, musculoskeletal disease, trauma, dementia, Parkinson’s disease, eye disease, ear disease, and other diseases. A physician or a health professional confirmed participants’ chronic disease history during the health examination.

IADL were evaluated using five items of instrumental self-maintenance, a subcategory of the 13-item Tokyo Metropolitan Institute of Gerontology Index of Competence [34]. Participants answered “yes” or “no” to the following five questions: “Can you use public transportation (bus or train) by yourself?”; “Are you able to shop for daily necessities?”; “Are you able to prepare meals by yourself?”; “Are you able to pay bills?”; and “Can you handle your own banking?”. Each item was scored 1 for “yes” and 0 for “no.” A score of 5 points indicated “independence,” and a score of ≤ 4 points showed “dependence” [35].

Mental health status was assessed using the Japanese version of the World Health Organization-Five Well-Being Index (WHO-5-J) [36]. The WHO-5-J is a self-administered questionnaire comprising five items. Item scores were summed to obtain a total score (range 0–25), with higher scores reflecting a higher level of subjective well-being. A score of ≥ 13 points indicated “no depression,” and a score of < 13 points indicated “depression.”

### Statistical analysis

We used the chi-square test to compare categorical variables and the *t*-test or analysis of variance to compare continuous variables. Multivariable logistic regression was used to determine the association between personality traits and polypharmacy. The multivariable logistic regression model was adjusted for the subjective economic situation, alcohol consumption and smoking habits, BMI, chronic diseases, IADL, and mental health status. Since there are sex differences between personality traits [37] and polypharmacy [3], separate analyses were performed for men and women. Multicollinearity (variance inflation factor) is less than 1.5 for each variable, for both men and women. The odds ratio per 0.5 SD, which corresponds to the minimal important difference [38] or each personality trait score, was also calculated.

All statistical analyses were performed using IBM SPSS 25.0 for Windows (IBM Corp., Armonk, NY, USA). A two-tailed *p*-value of < 0.05 was considered statistically significant.

### Ethical considerations

The study protocol was approved by the Institutional Review Boards of Osaka University Graduate Schools of Medicine, Dentistry, and Human Sciences and the Ethics Committee of Tokyo Metropolitan Institute of Gerontology. All participants provided written informed consent to participate.

## Results

The reliability of each domain of personality traits in this analysis was relatively high, with Cronbach’s alpha coefficients for neuroticism, extraversion, openness, agreeableness, and conscientiousness of 0.74, 0.75, 0.58, 0.70, and 0.73, respectively. The characteristics of the participants are provided in Table 1. Overall, 90.8% of participants were functionally independent. The mean ± standard deviation (SD) of the number of medications was 2.8 ± 2.8 in men and 3.1 ± 3.2 in women. The prevalence of polypharmacy was 23.9% and 28.0% in men and women, respectively. The mean scores (± SD) for neuroticism were 20.7 ± 5.3 in men and 22.3 ± 5.5 in women, those for extraversion were 26.6 ± 5.2 in men and 26.5 ± 5.2 in women, those for openness were 26.3 ± 4.3 in men and 27.2 ± 4.4 in women, those for agreeableness were 30.6 ± 4.2 in men and 32.7 ± 4.4 in women, and those for conscientiousness were 30.0 ± 4.7 in men and 30.0 ± 5.1 in women.

**Table 1:** Participant characteristics

	Men	Women	*P*-value^†^
	(*N* = 397)	(*N* = 439)	
Subjective economic status (%)			0.679
Economically difficult	96 (24.2)	96 (21.9)	
Usually adequate	213 (53.7)	238 (54.2)	
Economically adequate	88 (22.2)	105 (23.9)	
Drinking habits (current; %)	258 (65.0)	54 (12.3)	< 0.001
Smoking habits (current; %)	66 (16.6)	12 (2.7)	< 0.001
Body mass index (≥ 25; %)	91 (22.9)	95 (21.6)	0.656
IADL (dependence; %)	71 (17.9)	6 (1.4)	< 0.001
Depressive mood (depressive; %)	73 (18.4)	89 (20.3)	0.491
Number of chronic diseases (%)			0.077
0–1	140 (35.3)	124 (28.2)	
2	101 (25.4)	116 (26.4)	
≥ 3	156 (39.3)	199 (45.3)	
Number of medications (mean ± SD)	2.8 ± 2.8	3.1 ± 3.2	0.131
Polypharmacy (≥ 5; %)	95 (23.9)	123 (28.0)	0.179
Personality traits (mean ± SD)			
Neuroticism	20.7 ± 5.3	22.3 ± 5.5	< 0.001
Extraversion	26.2 ± 5.3	26.6 ± 5.2	0.268
Openness	26.3 ± 4.3	27.2 ± 4.4	0.003
Agreeableness	30.6 ± 4.2	32.7 ± 4.4	< 0.001
Conscientiousness	30.0 ± 4.7	30.0 ± 5.1	0.958

*IADL* instrumental activities of daily living, *SD* standard deviation

^†^ Tested using the chi-square test or t-test

Table 2 shows the characteristics of the participants with and without polypharmacy according to sex. In the comparison of the characteristics of participants with and without polypharmacy, women with polypharmacy were significantly more likely to be in an "economically difficult" situation than women without polypharmacy (*p* < 0.05). Women with polypharmacy were significantly less likely to have a drinking habit (*p* < 0.01). Both men and women with polypharmacy were significantly more likely to have three or more chronic conditions (both *p* < 0.001).

**Table 2:** Characteristics of participants with and without polypharmacy

	Men			Women		
	No Polypharmacy	Polypharmacy	*P* -value^†^	No Polypharmacy	Polypharmacy	*P* -value^†^
	(*N* = 312)	(*N* = 98)		(*N* = 331)	(*N* = 125)	
Subjective economic status (%)			0.051			0.031
Economically difficult	80 (26.5)	16 (16.8)		59 (18.7)	37 (30.1)	
Usually adequate	152 (50.3)	61 (64.2)		180 (57.0)	58 (47.2)	
Economically adequate	70 (23.2)	18 (18.9)		77 (24.4)	28 (22.8)	
Drinking habits (current; %)	200 (66.2)	58 (61.1)	0.357	47 (14.9)	7 (5.7)	0.009
Smoking habits (current; %)	51 (16.9)	15 (15.8)	0.802	7 (2.2)	5 (4.1)	0.286
Body mass index (≥ 25; %)	65 (21.5)	26 (27.4)	0.237	64 (20.3)	31 (25.2)	0.258
IADL (dependent; %)	52 (17.2)	19 (20.0)	0.537	3 (0.9)	3 (2.4)	0.227
Depressive mood (depressive; %)	52 (17.2)	21 (22.1)	0.284	57 (18.0)	32 (26.0)	0.062
Number of chronic diseases (%)			< 0.001			< 0.001
0–1	127 (42.1)	13 (13.7)		115 (36.4)	9 (7.3)	
2	88 (29.1)	13 (13.7)		92 (29.1)	24 (19.5)	
≥ 3	87 (28.8)	69 (72.6)		109 (34.5)	90 (73.2)	
Personality traits (mean ± SD)						
Neuroticism	20.4 ± 5.4	21.8 ± 4.9	0.020	21.9 ± 5.4	23.2 ± 5.6	0.021
Extraversion	26.3 ± 5.2	26.0 ± 5.6	0.686	27.0 ± 5.2	25.6 ± 5.1	0.012
Openness	26.4 ± 4.4	26.0 ± 3.9	0.424	27.2 ± 4.3	27.5 ± 4.6	0.483
Agreeableness	30.5 ± 4.2	30.6 ± 4.1	0.934	32.9 ± 4.4	32.4 ± 4.5	0.376
Conscientiousness	30.1 ± 4.8	29.8 ± 4.6	0.640	30.1 ± 5.3	30.0 ± 4.7	0.839

*IADL* instrumental activities of daily living, *SD* standard deviation

^†^ Tested using the chi-square test or t-test

As presented in Table 3, multivariable logistic regression analysis showed that neuroticism (adjusted odds ratio [aOR] per 1 point increase = 1.078, 95% confidence interval [CI] = 1.015–1.144) was significantly associated with polypharmacy in men, while extraversion (aOR = 0.932, 95% CI = 0.884–0.983) was significantly associated with polypharmacy in women. Additionally, the odds ratio per 0.5 SD, which corresponds to minimal important difference for each personality trait score, was 1.223 (95% CI = 1.041–1.434) for neuroticism in men and 0.831 (95% CI = 0.722–0.956) for extraversion in women.

**Table 3:** Associations between personality traits and polypharmacy

	Men (*N* = 397)		Women (*N* = 439)	
	aOR (95% CI)	*P*-value	aOR (95% CI)	*P*-value
Subjective economic status				
Economically difficult	ref		ref	
Usually adequate	0.511 (0.221–1.184)	0.117	1.475 (0.734–2.966)	0.275
Economically adequate	1.347 (0.687–2.643)	0.386	0.780 (0.428–1.421)	0.416
Drinking habits	0.564 (0.323–0.983)	0.043	0.398 (0.159–0.995)	0.049
Smoking habits	1.106 (0.532–2.297)	0.788	1.879 (0.450–7.855)	0.387
Body mass index	1.549 (0.847–2.836)	0.156	1.021 (0.585–1.784)	0.941
IADL	0.964 (0.484–1.921)	0.917	1.107 (0.175–7.005)	0.914
Depressive mood	1.197 (0.578–2.478)	0.629	1.162 (0.622–2.170)	0.639
Number of chronic diseases				
0–1	ref		ref	
2	1.377 (0.592–3.202)	0.457	3.730 (1.605–8.667)	0.002
≥ 3	8.680 (4.376–17.218)	< 0.001	11.60 (5.382–25.006)	< 0.001
Personality				
Neuroticism	1.078 (1.015–1.144)	0.015	1.023 (0.975–1.072)	0.357
Extraversion	1.009 (0.952–1.071)	0.757	0.932 (0.884–0.983)	0.010
Openness	0.988 (0.926–1.055)	0.719	1.049 (0.990–1.112)	0.107
Agreeableness	1.047 (0.976–1.123)	0.200	0.986 (0.927–1.048)	0.645
Conscientiousness	1.016 (0.956–1.080)	0.617	1.038 (0.985–1.094)	0.159

*aOR *adjusted odds ratio, *CI* confidence interval

The model adjusted for subjective economic status (economically difficult [reference]/usually/economically), drinking (no drinking [reference]/currently), smoking (not smoking [reference]/currently), body mass index (<25 kg/m2 [reference]/≥25 kg/m2 [obesity]), number of chronic diseases (0–1 [reference]/2/≥ 3), instrumental activities of daily living (independent [reference]/dependent), and depressive mood (none [reference]/depressive)

## Discussion

The results showed that higher levels of neuroticism and lower levels of extraversion were significantly associated with polypharmacy in men and women, respectively. The odds ratio per 0.5 SD for each personality trait score provided similar results.

This study showed that higher neuroticism was associated with polypharmacy in men. Neuroticism is characterized by emotional anxiety and vulnerability to stress. Neuroticism may influence lifestyle and lead to susceptibility to disease as a result. Previous studies have shown that higher levels of neuroticism are associated with smoking [39] and physical inactivity [40]. Further, consumption of more grains and dairy products, fish, vegetables, and fruits, has been associated with lower levels of neuroticism [41]. Thus, personality traits affect the lifestyles, resulting in unhealthy lifestyles that can lead to increased disability and increased mortality. Furthermore, personality traits may affect particular illnesses that require more medications, such as depression and diabetes mellitus. According to the studies on personality traits and diseases, higher levels of neuroticism are associated with depression [42], diabetes mellitus [25], and mild cognitive impairments [27]. Therefore, people with higher levels of neuroticism may be more prone to polypharmacy. Moreover, personality traits may affect attitude or thinking and behavior during illness. A previous study showed that regardless of the severity of anxiety and depression, patients with higher levels of neuroticism were more likely to have a perceived need for care [30]. ten Have et al. [43] have reported that people with high levels of neuroticism were more likely to receive care in the specialized mental health sector and visit healthcare services. Thus, people with higher levels of neuroticism may be more likely to report many symptoms, and this may prompt their doctors to prescribe many medications.

This study also showed that lower levels of extraversion were associated with polypharmacy in women. Extraversion is characterized by gregariousness, active and positive emotions. There have been studies on the relationship between extraversion and lifestyle. Previous studies have shown that lower levels of extraversion were negatively associated with healthy aware diet [41]. Previous studies have also shown that lower levels of extraversion were associated with physical inactivity [40, 44]. Moreover, extraverted people are likely to have healthier behaviors and better health, which may result in more prolonged survival [45]. Thus, personality traits can affect lifestyles, resulting in unhealthy lifestyles that can lead to increased disability and mortality. Furthermore, it is possible that personality traits with lower levels of extraversion affect a particular illness requiring many medications. Previous studies have shown that lower levels of extraversion are related to depression [42, 46] and infectious disease [28]. In addition, higher levels of extraversion have a protective effect against death from respiratory disease [47]. Although no data were shown in this study, there was a negative correlation between extraversion and lung disease in women, consistent with the findings of previous studies [47]. Moreover, personality traits with lower levels of extraversion may affect attitude or thinking and behavior during illness. As the characteristics of low levels of extraversion include the tendency to be reserved, retiring, and withdrawn [22], individuals with low levels of extraversion may not communicate their health problems to health care professionals. Lack of communication between healthcare professionals and patients can interfere with an accurate diagnosis by healthcare professionals, leading to multidrug prescriptions and multiple medical treatments. Additionally, in previous studies, extraversion was associated with social relationships [48] and positively correlated with affectionate support [49]. Therefore, people with lower levels of extraversion are poorly supported, and this can make their medical conditions more serious, leading to multiple drug use.

The results of this study suggest that personality traits may affect the development of illness and medication usage (polypharmacy). However, the causal pathway by which personality traits are related to polypharmacy is unclear. There are several possible mechanisms by which personality traits may lead to polypharmacy; these include: (1) personality traits may influence lifestyle, and an unhealthy lifestyle makes individuals more prone to disease; (2) personality traits may influence the manifestation and severity of a specific illness, thus influencing the amount of medication that is required; and (3) personality traits may affect thinking and behavior patterns during illness, which may affect the use of medical services, treatment needs, and communication with doctors.

This study identified a sex difference, in that different personality traits were associated with polypharmacy. Furthermore, the background between personality and polypharmacy may have different pathways depending on sex. Because, in addition to body composition, organ, and metabolism, there are sex differences in lifestyles, such as drinking, smoking, physical activity [50], dietary intake [51], and factors leading to long-term care [52]. In addition to the occurrence of sex-specific morbidities due to the above factors, personality-specific medical treatment behaviour may have affected the number of medications taken. Sex differences need to be examined in detail in the future.

In addition to personality traits, not having a drinking habit and having a high number of chronic diseases were independently associated with polypharmacy. Similar to the present study, a previous study reported that having a drinking habit was inversely associated with polypharmacy; that is, an increase in the frequency of alcohol consumption led to a decrease in polypharmacy [18]. Although a causal relationship cannot be claimed, this being a cross-sectional study, it was estimated that people with chronic diseases may be more likely to quit drinking. It has also been reported that a high number of chronic diseases was associated with polypharmacy [3], which is consistent with the present study.

This study has some strengths. First, personality traits were based on a self-reported questionnaire. In this study, the NEO-FFI was used to evaluate personality traits. The NEO-FFI was developed by Costa et al. [32], and the reliability and validity of the Japanese version of the NEO-FFI have been verified by Shimonaka et al. [33]. The NEO-FFI is a stable and standard measure and is often used in gerontological studies. In addition, data on medication status were also collected through interviews. To minimize the inaccuracy of the medication status due to self-reports, we asked participants to bring their medication records. Based on data in this record, the physician confirmed the medication status in face-to-face interviews. Therefore, the number of prescriptions was accurate. Second, a limited number of studies have examined the relationship between personality traits and polypharmacy in older adults. Some studies on personality traits and polypharmacy have focused on patients with impaired mental health. A previous study on older adults with mental illness found that neuroticism was associated with polypharmacy [23]. In addition, low levels of openness, extraversion, and conscientiousness have been reported to be associated with increased psychotropic medication use in patients with bipolar disorder [53]. This study provides new findings regarding the relationship between personality traits and polypharmacy in community-dwelling older adults.

However, this study has some limitations. First, there is a possibility of residual confounding. The results showed that personality traits were associated with polypharmacy. The variables adjusted in the analysis of the relationship between personality traits and polypharmacy included economic status as socio-economic factors; drinking and smoking status as lifestyle-related factors; and BMI, IADL, mental health, and the number of chronic diseases as health status factors. Even after adjusting for various aspects of health status, the association remained significant, so poor health status did not completely explain the number of medications prescribed to older adults. In addition, since mental illness measures were not included in the medical history, the WHO-5-J was used as an adjusting variable in the model. Therefore, the effects of mental illness were considered adjustable. Furthermore, although the data are not presented in this study, neuroticism in men and extroversion in women remain significantly associated with polypharmacy after adjusting for psychiatric drug use. However, although several factors were controlled for in the analyses, residual confounders, such as social support and social network, might be present. It has been reported that social support is associated with better self-care compliance among older patients [54]. In addition, inclusion in social networks is associated with healthy behavior [55] and a reduced risk of depressive symptoms [56]. Therefore, if the analysis is adjusted for social factors, the strength of association between personality traits and polypharmacy may be weakened.

Second, the representativeness may be limited. The participation rate of this study was 23.2%, which is a low rate. The participants had at least a degree of independence as they could attend the study venue independently. Most participants were physically and cognitively healthy; therefore, people with poor health status may not have participated in this study. Conversely, most individuals in their 70 s are independent and active. It is possible that the low participation rate in the survey was because potential participants were engaged in work or leisure activities.

Third, there were cultural differences. The participants in this study were older adults living in Japan. Therefore, attention should be paid when generalizing the study results to other countries with different medical systems and cultural backgrounds. It is necessary to consider whether personality traits are commonly associated with polypharmacy, even in older adults with diverse backgrounds.

Fourth, a causal relationship was not described. Because the study was a cross-sectional study, our findings could not identify causal relationships. However, personality traits influence lifestyle [24, 40, 44] and behavior [30, 43]. A previous study of depressed patients found that the use of medication decreased neuroticism, increased extroversion and reduced depression [57]. However, these results are inconsistent with the results of our study. Approximately 50% of personality traits are attributable to additive genetic effects [58], and personality is stable after 30 years of age [32]. Therefore, the effect of polypharmacy on personality is likely to be small. Longitudinal research is required to clarify causal relationships in the future.

Nevertheless, our results suggest that personality traits may be involved in the process of disease outbreaks or behaviors toward treatment. The relationship between personality traits and various emotional reactions and behaviors is a common phenomenon. Therefore, understanding the personality traits of individuals will be helpful in effective treatment and health interventions in independent community-dwelling older adults. In particular, personality traits may be beneficial in the prevention and management of unhealthy lifestyles, such as unbalanced dietary habits, physical inactivity, smoking, and lifestyle-related diseases caused by unhealthy lifestyles. More specifically, information on the patient’s personality traits may contribute to (1) diagnosis that considers unhealthy lifestyles and illness trends, (2) treatment plan that considers the patient’s request and attitude toward treatment, communication style, and support status, and (3) intervention design suitable for each personality trait, such as group and individual interventions.

The study results showed that higher levels of neuroticism and lower levels of extraversion in men and women, respectively, are associated with polypharmacy. Therefore, to avoid polypharmacy, it is necessary to prevent and improve unhealthy habits and mental health deterioration in men with higher levels of neuroticism or women with lower levels of extraversion. In addition, since patients with higher levels of neuroticism tend to require more medications and treatments, it is necessary to control anxiety and prevent excessive medical care in patients. Furthermore, since lower levels of extraversion involve poor communication or support, it is necessary to understand patients' symptoms by creating an environment that enhances communication and provides care considering the support environment of the patients.

## Conclusions

In conclusion, this cross-sectional study showed that personality traits, especially higher levels of neuroticism in men and lower levels of extraversion in women, were associated with polypharmacy, even after adjusting for potential confounding factors. This study suggests that personality traits may affect medication usage in older adults and need to be considered in managing polypharmacy.

## Data Availability

The datasets generated and/or analysed during the current study are not publicly available due to privacy/ethical restrictions but are available from the corresponding author on reasonable request.

## Abbreviations

aOR: Adjusted odds ratio
BMI: Body Mass Index
CI: Confidence interval
IADL: Instrumental of activity if daily living
NEO-FFI: The NEO-Five-Factor Inventory
SD: Standard deviation
SONIC: Septuagenarians, Octogenarians, and Nonagenarians Investigation with Centenarians
WHO-5-J: The World Health Organization-Five Well-Being Index
